# Patients prefer easy adverse event reporting: Observational study within clinical trial

**DOI:** 10.1177/20552076251345894

**Published:** 2025-05-25

**Authors:** Lauri Lukka, Maria Vesterinen, Joonas J. Juvonen, Satu Palva, J. Matias Palva

**Affiliations:** 1 Department of Neuroscience and Biomedical Engineering, 174277 Aalto University, Espoo, Finland; 2 Neuroscience Center, Helsinki Institute of Life Science, 3835 University of Helsinki, Helsinki, Finland; 3 Centre for Cognitive Neuroimaging, School of Psychology and Neuroscience, 3526 University of Glasgow, Glasgow, UK

**Keywords:** Adverse events, clinical trials, depression, digital interventions, mental health, mixed methods, monitoring, safety evaluation

## Abstract

**Background:**

Digital intervention safety is crucial for regulatory approval and clinical adoption. However, the evaluation and reporting of adverse events (AEs) in clinical trials are often insufficient. Digital qualitative self-reporting could enhance the detection of AEs, but patient preferences for using such channels remain understudied.

**Methods:**

This observational study was conducted in Finland between 2022 and 2024 within a randomized controlled trial evaluating the efficacy of *Meliora*, a game-based digital intervention for patients living with major depressive disorder. We assessed the preferences of 1001 patients for self-reporting AEs across four channels: a prompted, within-intervention questionnaire (CORTO: Contextual, One-item, Repeated, Timely, Open-ended), a Jira questionnaire, email, and phone.

**Results:**

148 (14.8%) patients reported AEs during the study. We found a significant imbalance between the channels: 11.3% (*n* = 113) of patients reported AEs using CORTO, 4.1% (*n* = 41) using email, 1.1% (*n* = 11) using Jira, and 0.4% (*n* = 4) using phone.

**Conclusions:**

These findings reveal that patients prefer low-effort methods for reporting AEs and are more likely to report AEs via a prompted, within-intervention questionnaire (CORTO) than through other methods. Integrating qualitative self-report channels into digital interventions may enhance AE detection rates, improve clinical trial safety monitoring, and support post-market surveillance.

## Introduction

Patients living with mental disorders face substantial gaps in treatment access^1,2^ and effectiveness.^3^ Novel digital therapeutic devices are actively developed^4^ to mitigate these problems. Human-supported digital mental health interventions (DMHIs) already achieve outcomes comparable to face-to-face treatments^5,6^ and next-generation interventions—such as game-based DMHIs—could further improve intervention reach, engagement, and efficacy.^7–9^ Medical device manufacturers must provide evidence of effectiveness and safety prior to market access^10–12^ and must also engage in post-market surveillance to ensure proper device functioning and performance.^11,13^ These data allow patients, clinicians, and decision-makers to weigh the benefits of an intervention against its potential risks. However, many clinical trials collect, analyze, and report adverse events (AEs) insufficiently or inconsistently.^12,14,15^ Information on AEs thus remains unpublished^16^ despite the Consolidated Standards for Reporting Trials^17^ and its 2022 Harms update,^12^ which advocate comprehensive reporting.

Although many digital interventions have a low risk profile, patients still report AEs, including symptom deterioration, new symptoms, and frustration or stress related to intervention use. For instance, 9.3% of 558 patients in four clinical trials reported negative effects.^18^ However, AE reporting remains inconsistent. One review found that only 6 of 23 (26%) trials evaluating DMHIs reported AEs,^19^ while another review identified AE reporting in just 55 of 171 (32%) mental health app trials.^20^ Another review, examining digital interventions for psychosis, found that 16 of 34 shared datasets reported no AEs, while the remaining 18 datasets reported 593 AEs in 1600 participants.^21^ These findings suggest either considerable variability in AE occurrence or an underestimation of AEs across interventions. There is thus a growing interest in improving the monitoring and reporting of AEs in randomized controlled trials (RCTs) evaluating digital interventions.^22,23^

AEs have been monitored using standardized questionnaires, diaries, interviews, and open-ended questions in trials investigating both traditional^14^ and digital interventions.^20,22,23^ Monitoring may be active or passive,^14,23^ and the frequency can vary greatly from daily to monthly intervals.^23^ Since clinical trials last several weeks or months, it is recommended that AEs are measured regularly.^22,23^ However, practical solutions for doing so have remained limited. The CORTO method (Contextual, One-Item, Repeated, Timely, Open-Ended)^24^ may offer a viable solution for frequent, within-intervention AE monitoring. This qualitative, prompted approach could help to mitigate recall issues^25^ that limit the accuracy and comprehensiveness of delayed data acquisition. However, it is still unclear whether patients prefer using such a channel over more conventional alternatives.

In this study, we asked which channels patients prefer for self-reporting AEs. This observational study compared the frequency of patients using CORTO,^24^ Jira-based questionnaire, email, and phone call.

## Methods

This observational study investigated which channels patients used to self-report AEs. It was conducted within a pre-registered^26^ (NCT05426265), double-blinded, comparator-controlled trial, *Meliora RCT*, conducted in Finland. The trial evaluated the efficacy of *Meliora*, a novel game-based DMHI,^7^ intended to alleviate symptoms of major depressive disorder (MDD). The study was approved by the Helsinki University Hospital (HUS) Regional Committee on Medical Research Ethics (HUS/3042/2021) and the Finnish Medicines Agency (FIMEA/2022/002976). Informed consent to participate was obtained digitally from all participants. The STROBE checklist^27^ is included in Appendix 1.

### Patients

Eligible patients were Finnish-speaking adults aged 18 to 65 years living with MDD, which is characterized by low mood and loss of interest causing significant distress or impairing functioning.^28^ A total of 1384 patients were recruited between 28 June 2022 and 14 August 2024 via social media, healthcare partners, email campaigns, and posters.^29^ Clinical study coordinators (CSCs) remotely confirmed the MDD diagnoses using the Mini-International Neuropsychiatric Interview.^30^ Additionally, CSCs ensured each patient had an ongoing mental health treatment contact, good eyesight, access to a suitable computer, and a valid email address and phone number for communication during the study. CSCs also confirmed that patients did not meet the exclusion criteria of active suicidality, gaming addiction, psychotic or neurological disorders, pregnancy or nursing status, inability to provide consent, or current incarceration or forensic hospitalization. Patients accessed the intervention at home on their personal computers. They were instructed to engage with the intervention for a total of 48 h (minimum 24 h), during the 12-week intervention period.

### Measuring adverse events

Patients could self-report AEs using four channels: the CORTO questionnaire,^24^ a Jira questionnaire, email, and phone calls. The availability of these complementary channels was intended to encourage reporting, allowing patients to choose the method that best suited their preferences. These channels were pre-defined in the Clinical Investigation Plan.

Patients were informed about potential AEs through the informed consent form, intervention splash screen (a page briefly displayed at intervention startup before entering the main menu), and within-intervention safety page accessible from the main menu (Appendix 2). These sources advised patients to stop using the intervention if they experienced any AEs and encouraged them to report AEs via CORTO, the main menu button (leading them to the Jira questionnaire), or email (Table 1). CSCs actively monitored email, phone, and Jira daily to respond promptly to patient concerns. AEs were conceptualized as “*any untoward medical occurrence in a patient or clinical investigation subject administered a pharmaceutical product and which does not necessarily have a causal relationship with this treatment”*.^31^ If a patient reported multiple instances of the same AE, only one instance was counted.

**Table 1.** Characteristics of the adverse events reporting channels used in the study.

Channel	Description	Prompted	Within intervention	Within PC	Interactive
**CORTO**	Patient receives a questionnaire after each of 28 intervention levels.	✓	✓	✓	
**Jira**	Patient can access the user support questionnaire via the main menu at any time.		✓	✓	
**Email**	Patient can email the clinical subject coordinators.			✓	✓
**Phone**	Patient can call the clinical subject coordinators.				✓

Patients were prompted with the CORTO questionnaire each time they completed one of the 28 intervention levels (see Figure 3 in^24^). This questionnaire included one open-ended item where patients could provide feedback, describe their experience, and report any AEs in free text. During the intervention period, the time required to progress through the levels increased, which reduced the frequency of CORTO prompts.

The intervention's main menu included a “User Support” option (In Finnish: “Käyttäjätuki”), which linked to a browser-based questionnaire implemented in Jira (Atlassian Ltd). Patients could use this form to report bugs, request technical support, ask questions, provide feedback, and report AEs in free text (Appendix 3).

Patients could also contact CSCs via email and phone. The email address was available on the study website, sign-up forms, the informed consent form, symptom questionnaires, and the splash screen. It was also used to schedule the remote pre-intervention evaluation. Patients used their preferred email services on their own devices (e.g. computer, smartphone, or tablet) to report their experiences in free text. Additionally, patients could call CSCs using the phone number provided in the informed consent form, symptom questionnaires, and used for pre-intervention evaluation. CSCs documented patient reports with secure backend study management software.^31^

### Statistical analysis

For the main contrasts, a chi-square test with an alpha level of 0.05 was used to compare the frequencies of participants using each reporting channel across all channel pairs. The resulting *p*-values were subjected to False Discovery Rate correction using the Benjamini–Hochberg method. Confidence limits for the percentages of patients using each channel were obtained through bootstrapping the subpopulations of binary outcomes (10,000 resamples).

## Results

**Figure 1.**
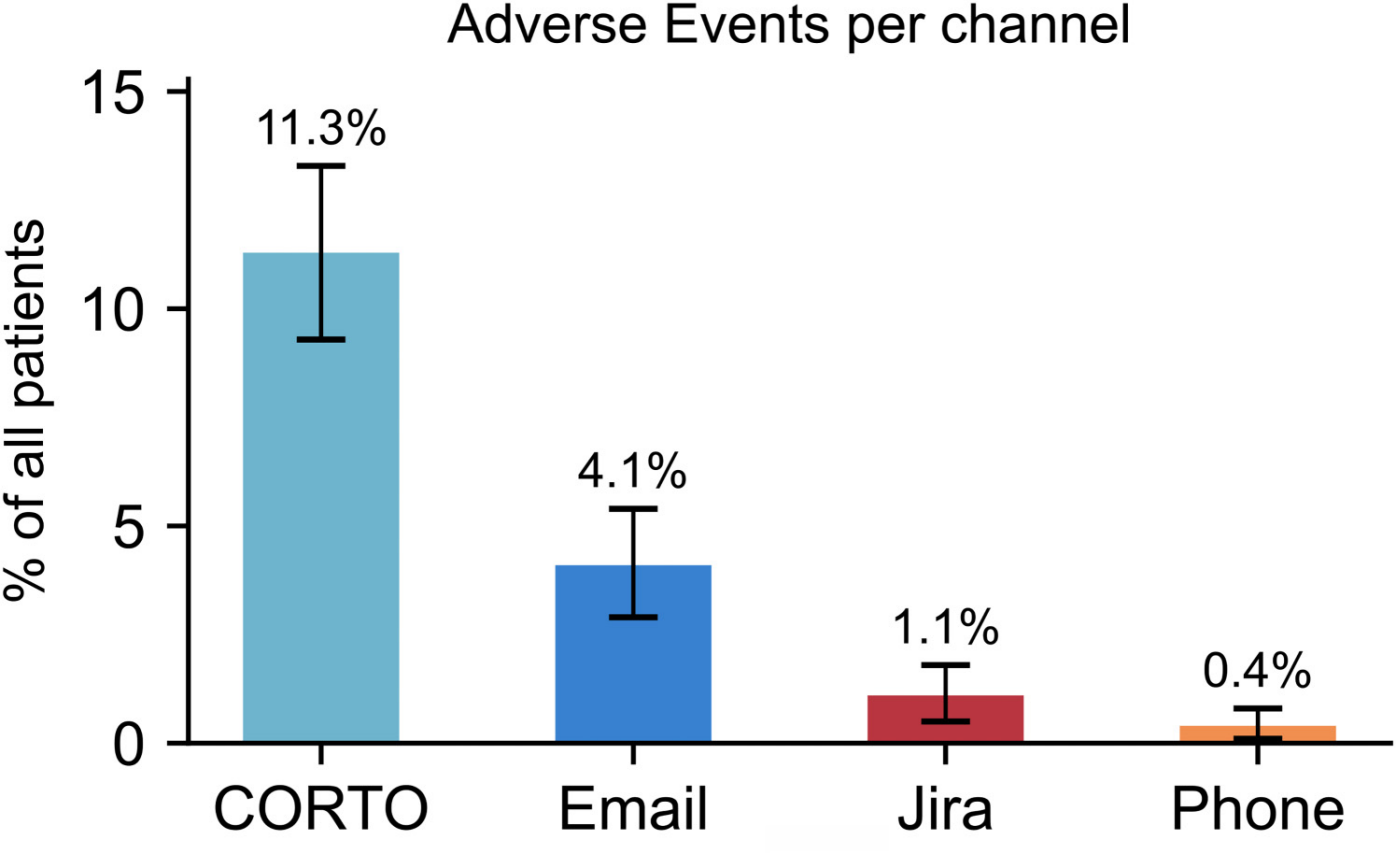
Proportion of all patients who reported at least one adverse event using CORTO, email, Jira, or phone.

A total of 1001 patients were accepted into *Meliora RCT* with an average age of 33.6 years (SD = 9.8) and 64.1% identifying as female. Of these, 14.8% (*n* = 148) reported one or more AEs. On average, patients who reported AEs used 1.1 ± 0.4 (mean ± SD) reporting channels. The most frequently used reporting channel was CORTO (11.3%, *n* = 113), followed by email (4.1%, *n* = 41), Jira (1.1%, *n* = 11), and phone (0.4%, *n* = 4) (Figure 1). Patients were significantly more likely to report AEs using CORTO than via email, Jira, or phone (Table 2). Email was used significantly more often than Jira or phone. However, there was no statistically significant difference between the use of Jira and phone.

**Table 2.** Chi-square test comparing channel usage frequencies. *p_raw_* indicates uncorrected *p*-values, *p_fdr_* indicates FDR-corrected *p*-values, and ** indicates significance at *p* < 0.05.

Channel	Email	Jira	Phone
**CORTO**	χ² = 35.46,	χ² = 87.70,	χ² = 105.88,
	*dof* = 1,	*dof* = 1,	*dof* = 1,
	*n* = 154,	*n* = 124,	*n* = 117,
	*p_raw_ =* 2.60 × 10^−9^**	*p_raw_ =* 7.63 × 10^−21^**	*p_raw_ =* 7.83 × 10^−25^**
	*p_fdr_* = 5.20 × 10^−9^**	*p_fdr_* = 2.29 × 10^−20^**	*p_fdr_* = 4.70 × 10^−24^**
**Email**		χ² = 16.60,	χ² = 29.46,
		*dof* = 1,	*dof* = 1,
		*n* = 52,	*n* = 45,
		*p_raw_ =* 4.60 × 10^−5^**	*p_raw_ =* 5.70 × 10^−8^**
		*p_fdr_* = 5.53 × 10^−5^**	*p_fdr_* = 8.55 × 10^−8^**
**Jira**			χ² = 2.42,
			*dof* = 1,
			*n* = 15,
			*p_raw_ =* .12
			*p_fdr_* = .12

## Discussion

This study revealed notable differences in how patients reported AEs across the four available channels. The most frequently used channel was CORTO (11.3%), a within-intervention questionnaire that was prompted multiple times throughout the intervention period. The second most common channel was email (4.1%) that was familiar to the patients, available on patients’ personal devices, used to manage trial processes, and used in communication with the CSCs. In contrast, reporting via the Jira questionnaire (1.1%) or phone (0.4%) was less prevalent, which may be attributable to that Jira was only accessible through a single menu button, while calling required direct social interaction and was only available during office hours. These findings suggest that patients are more likely to use a reporting channel the less effort it requires.

We found that 14.8% of patients using a game-based DMHI for MDD reported AEs when they were measured throughout the intervention period using four channels. For comparison, a previous study reported that 9.3% of patients using a digital cognitive behavioral therapy intervention experienced AEs when measured once post-intervention.^18^ Another study found that 22.7% of participants with self-reported depressive symptoms using a self-help smartphone application reported AEs when measured using a standardized inventory for assessing negative effects.^32^ The observed prevalence of AEs conceivably depends on the investigated intervention and the methods for measuring AEs.

Prompted digital self-reported methods, such as CORTO,^24^ present a new approach to address the need for repeated AE monitoring in clinical trials.^22,23^ In the present study, CORTO was by far the most frequently used channel, suggesting that this approach may facilitate the detection of AEs. This aligns with our previous findings, which showed that CORTO generated more specific user experience data than retrospective interviews.^24^ Moreover, as a qualitative self-report method, CORTO is capable of capturing both expected and unexpected harms.^12,15^ As Allan et al. note: “*Providing a way for patients to self-report AEs may increase the quality of information available to researchers and bring greater understanding around potential harms of the intervention under investigation”.*^21^ Importantly, our results suggest that not all qualitative self-report channels are equally effective, and that the channel design may influence how likely patients are to use them (see Table 1).

When conceptualizing deterioration or the emergence of novel symptoms as AEs, these constructs can be evaluated using standardized measures,^12^ such as depressive symptom severity using the PHQ-9.^33^ Future studies could explore the overlap between AEs detected in qualitative self-reports and those related to symptom deterioration captured by standardized measures.

Some patients may prefer channels that allow dialog. In this study, email was the second most frequently used channel after CORTO, and in a related study, we discovered that 31.1% of patients contacted the researchers via email.^29^ The most common were giving feedback and asking for technical support (13.1%), wishing to quit the study (6.7%), asking for technical support with the symptom questionnaires (6.3%), and resolving uncertainties regarding the study processes (6.2%), and among these contacts were also the AE reports. Channels that enable bidirectional communication (e.g. email or phone) may therefore remain essential for patients.

This study has several limitations. First, its observational design limits causal inference. Without a control group, it is unclear whether the patients would have used alternative channels in the absence of CORTO. While it is plausible that some patients might have used alternative reporting methods, the proportion of such cases cannot be determined from the present data. Second, we previously noted that CORTO may be particularly well-suited for interventions with extensive content used in naturalistic settings.^24^ Thus, its applicability to shorter interventions is uncertain and requires further investigation. Third, the sample consisted of Finnish-speaking patients living with depression (for detailed patient qualities, see^34^) which may limit generalizability to other populations. Fourth, this study focused exclusively on self-reported AEs and did not assess other possible negative effects such as symptom deterioration, treatment dropout, or nonresponse.^22^ These factors are important for understanding overall intervention safety. Finally, this study did not explore how the reported AEs could be used to mitigate identified risks^23^ or refine intervention content. Future work could examine how patient-reported qualitative data can inform iterative intervention design,^35,36^ thereby improving the quality, safety, and effectiveness of digital interventions.

## Conclusion

This study reveals that patients are more likely to report AEs using low-effort channels. Specifically, patients were more likely to report AEs using CORTO (11.3%) than via email (4.1%), the Jira questionnaire (1.1%), or phone (0.4%). These findings suggest that repeatedly prompted within-intervention self-report methods facilitate the reporting of AEs and generate more complete safety data in digital mental health trials and post-market surveillance.

## Figures and Tables

**Appendix Figure 1.**
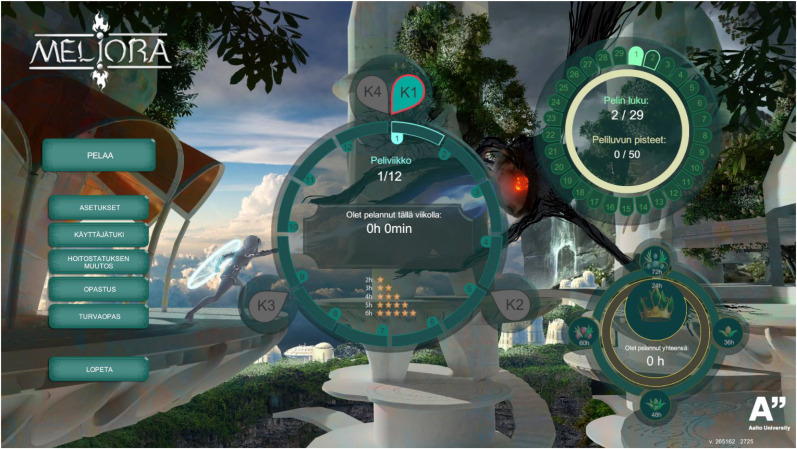
The main menu of the Meliora intervention. User support (In Finnish: “Käyttäjätuki”) allowed participants to access the Jira-based questionnaire. Safety Information (In Finnish: “Turvaopas”) allowed participants to access safety information.

**Appendix Figure 2.**
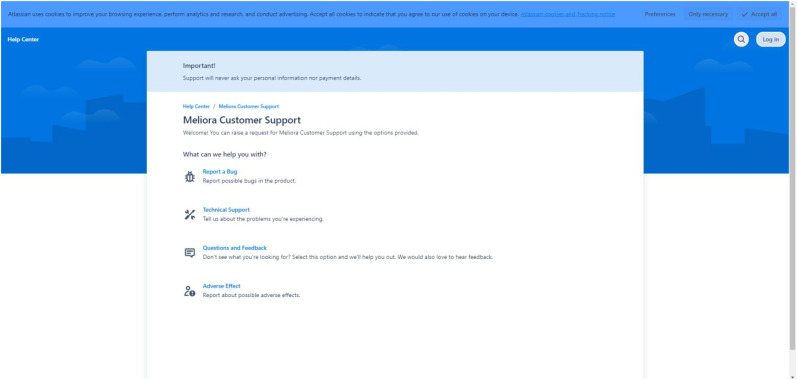
Clicking the “User support” button (see Figure 1) opened a browser-based reporting system, Jira. This system allowed the participant to report a bug, request technical support, ask questions and give feedback, and report adverse effects.

**Appendix Figure 3.**
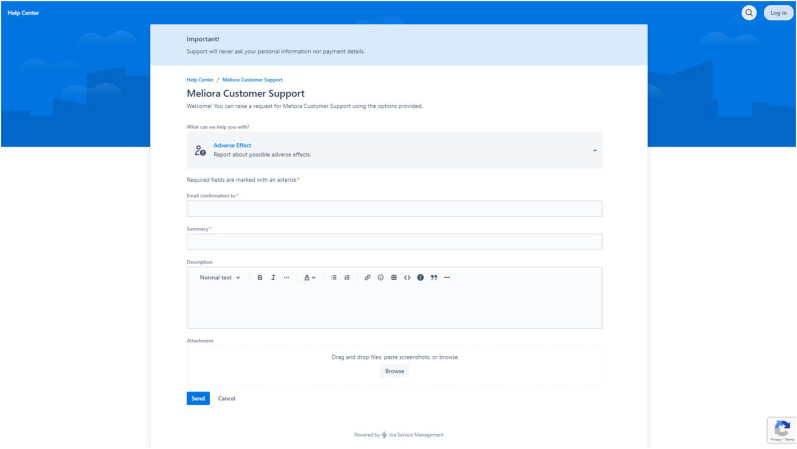
Choosing the last option (see Figure 2) opened the following questionnaire, which allowed reporting the adverse effects.

## Abbreviations

AEs: Adverse Events
CSCs: Clinical Subject Coordinator
FDR: False Discovery Rate
CORTO: Contextual, One-Item, Repeated, Timely, Open-Ended
MDD: Major Depressive Disorder
RCT: Randomized Clinical Trial

## Appendix 1. STROBE statement

STROBE Statement—checklist of items that should be included in reports of observational studies

**Table:**

	Item No	Recommendation	Page No
**Title and abstract**	1	(*a*) Indicate the study's design with a commonly used term in the title or the abstract	1
		(*b*) Provide in the abstract an informative and balanced summary of what was done and what was found	1
Introduction			
Background/rationale	2	Explain the scientific background and rationale for the investigation being reported	1–2
Objectives	3	State specific objectives, including any prespecified hypotheses	2
Methods			
Study design	4	Present key elements of study design early in the paper	2–3
Setting	5	Describe the setting, locations, and relevant dates, including periods of recruitment, exposure, follow-up, and data collection	2
Participants	6	(*a*) *Cohort study*—Give the eligibility criteria, and the sources and methods of selection of participants. Describe methods of follow-up *Case-control study*—Give the eligibility criteria, and the sources and methods of case ascertainment and control selection. Give the rationale for the choice of cases and controls *Cross-sectional study*—Give the eligibility criteria, and the sources and methods of selection of participants	2
		(*b*) *Cohort study*—For matched studies, give matching criteria and number of exposed and unexposed *Case-control study*—For matched studies, give matching criteria and the number of controls per case	-
Variables	7	Clearly define all outcomes, exposures, predictors, potential confounders, and effect modifiers. Give diagnostic criteria, if applicable	2–3
Data sources/ measurement	8*	For each variable of interest, give sources of data and details of methods of assessment (measurement). Describe comparability of assessment methods if there is more than one group	2–3
Bias	9	Describe any efforts to address potential sources of bias	2–3
Study size	10	Explain how the study size was arrived at	2–3
Quantitative variables	11	Explain how quantitative variables were handled in the analyses. If applicable, describe which groupings were chosen and why	3
Statistical methods	12	(*a*) Describe all statistical methods, including those used to control for confounding	3
		(*b*) Describe any methods used to examine subgroups and interactions	3
		(*c*) Explain how missing data were addressed	2–3
		(*d*) *Cohort study*—If applicable, explain how loss to follow-up was addressed *Case-control study*—If applicable, explain how matching of cases and controls was addressed *Cross-sectional study*—If applicable, describe analytical methods taking account of sampling strategy	-
		(*e*) Describe any sensitivity analyses	-

**Table:**

Results			
Participants	13*	(a) Report numbers of individuals at each stage of study—eg numbers potentially eligible, examined for eligibility, confirmed eligible, included in the study, completing follow-up, and analysed	2–3
		(b) Give reasons for non-participation at each stage	3
		(c) Consider use of a flow diagram	-
Descriptive data	14*	(a) Give characteristics of study participants (eg demographic, clinical, social) and information on exposures and potential confounders	3
		(b) Indicate number of participants with missing data for each variable of interest	-
		(c) *Cohort study*—Summarise follow-up time (eg, average and total amount)	-
Outcome data	15*	*Cohort study*—Report numbers of outcome events or summary measures over time	3–4
		*Case-control study—*Report numbers in each exposure category, or summary measures of exposure	
		*Cross-sectional study—*Report numbers of outcome events or summary measures	
Main results	16	(*a*) Give unadjusted estimates and, if applicable, confounder-adjusted estimates and their precision (eg, 95% confidence interval). Make clear which confounders were adjusted for and why they were included	3–4
		(*b*) Report category boundaries when continuous variables were categorized	-
		(*c*) If relevant, consider translating estimates of relative risk into absolute risk for a meaningful time period	-
Other analyses	17	Report other analyses done—eg analyses of subgroups and interactions, and sensitivity analyses	-
Discussion			
Key results	18	Summarise key results with reference to study objectives	3
Limitations	19	Discuss limitations of the study, taking into account sources of potential bias or imprecision. Discuss both direction and magnitude of any potential bias	5
Interpretation	20	Give a cautious overall interpretation of results considering objectives, limitations, multiplicity of analyses, results from similar studies, and other relevant evidence	3–5
Generalisability	21	Discuss the generalisability (external validity) of the study results	3–5
Other information			
Funding	22	Give the source of funding and the role of the funders for the present study and, if applicable, for the original study on which the present article is based	6

*Give information separately for cases and controls in case–control studies and, if applicable, for exposed and unexposed groups in cohort and cross-sectional studies.

**Note:** An Explanation and Elaboration article discusses each checklist item and gives methodological background and published examples of transparent reporting. The STROBE checklist is best used in conjunction with this article (freely available on the Web sites of PLoS Medicine at http://www.plosmedicine.org/, Annals of Internal Medicine at http://www.annals.org/, and Epidemiology at http://www.epidem.com/). Information on the STROBE Initiative is available at www.strobe-statement.org.

## Appendix 2. Safety information

### Informed consent form

This information was provided to the participant in the informed consent form.

### Original, in Finnish

Tutkimuksen hyödyt, vaikutukset, riskit ja haitat

Tutkimuksessa tutkitaan peliä, jonka tavoite on vaikuttaa lieventävästi masennuksen oireisiin. Tutkimuksesta ei kuitenkaan voida olettaa olevan tutkittavalle selvää lääketieteellistä hyötyä.

Jotkut ihmiset ovat herkkiä välkkyville valoille tai kuvioille, ja he voivat saada niistä epilepsiakohtauksia. Et voi osallistua tutkimukseen, jos sinulla on epilepsia tai olet saanut epileptisiä kohtauksia.

Pelin mahdollisia haittavaikutuksia ovat turhautuminen, tylsistyminen, stressi, silmäsärky, päänsärky, huimaus, pahoinvointi, väsymys, niskakivut, näköhäiriöt, migreeni, lihasten nykiminen, epämukavat tuntemukset tai kipu esimerkiksi käsissä, ja ajan- ja paikantajun menetys. Jos koet haittoja pelaamisesta, lopeta pelin pelaaminen. Mahdolliset haittavaikutukset tulee raportoida tutkijoille pelin sisällä olevan kaavakkeen kautta.

Suosittelemme huomioimaan hyvän ergonomian, riittävän valaistuksen ja tauot pelaamisen aikana.

### Translated, in English

#### Benefits, effects, risks, and adverse events of the study

This study investigates a game that aims to alleviate the symptoms of depression. However, it cannot be assumed that the study will provide clear medical benefits.

Some people are sensitive to flashing lights or patterns, which can trigger epileptic seizures. You cannot participate in the study if you have epilepsy or have experienced epileptic seizures.

Possible adverse events of the game include frustration, boredom, stress, eye strain, headache, dizziness, nausea, tiredness, neck pain, visual disturbances, migraine, muscle twitching, discomfort or pain (e.g. in the hands), and loss of sense of time and place. If you experience any adverse events while playing, stop using the game. Any adverse events should be reported to the researchers via the in-game form.

We recommend practicing good ergonomics, ensuring adequate lighting, and taking breaks while playing.

### Splash screen

This screen was shown every time the intervention was launched.

### Translated, in English

#### Light-sensitive epilepsy

If you have epilepsy or you have experienced epileptic seizures, you cannot participate in this study. Some people are sensitive to flashing lights and geometric shapes and patterns, or they may have latent epilepsy. They may experience epileptic seizures while playing games or watching videos even if they have not had such seizures previously.

#### Possible harms

Possible adverse effects of the game include frustration, boredom, stress, eye strain, headache, dizziness, nausea, tiredness, neck pain, visual disturbances, migraine, muscle twitching, discomfort or pain (e.g. in the hands), and loss of sense of time and place.

If you experience harms, discontinue playing the game. If the harm continues, contact a medical professional.

#### Reporting harms

If you experience harms from playing, report them via the “User support” button. You can also contact the researchers via email (meliora@aalto.fi).

#### Use and handling

Play in a well-lit environment and maintain an adequate distance from the computer screen. Remember good ergonomics and playing posture while playing. You can play Meliora for a maximum of 90 min per day. Keep enough breaks while playing. Avoid playing when you are tired or suffer from lack of sleep.

### Safety information

This Safety Information was accessible within the intervention main menu.

### Translated, in English

#### Light-sensitive epilepsy

If you have epilepsy or you have experienced epileptic seizures, you cannot participate in this study. Some people are sensitive to flashing lights and geometric shapes and patterns, or they may have latent epilepsy. They may experience epileptic seizures while playing games or watching videos even if they have not had such seizures previously.

#### Possible harms

Possible adverse effects of the game include

- Frustration
- Boredom
- Stress
- Eye strain
- Headache
- Dizziness
- Nausea
- Tiredness
- Neck pain
- Visual disturbances
- Migraine
- Muscle twitching
- Discomfort or pain (e.g., in the hands)
- Loss of sense of time and place.

#### Reporting harms

If you experience harms from playing, report them via the “User support” button.

You can also contact the researchers via email (meliora@aalto.fi).

#### Use and handling

Play in a well-lit environment and maintain an adequate distance from the computer screen. Remember good ergonomics and playing posture while playing.

You can play Meliora for a maximum of 90 min per day. Keep enough breaks while playing.

Avoid playing when you are tired or suffer from lack of sleep.
